# Renal protective effects of Alpinate Oxyphyllae Fructus and mesenchymal stem cells co-treatment against D- galactose induced renal deterioration

**DOI:** 10.7150/ijms.96007

**Published:** 2024-05-27

**Authors:** Tsung-Jung Ho, Tamilselvi Shanmugam, Po-Hsiang Liao, Marthandam Asokan Shibu, William Shao-Tsu Chen, Kuan-Ho Lin, Shang-Yeh Lu, Chia-Hua Kuo, Wei-Wen Kuo, Chih-Yang Huang

**Affiliations:** 1 Department of Chinese Medicine, Hualien Tzu Chi Hospital, Buddhist Tzu Chi Medical Foundation, Hualien, Taiwan.; 2 Integration Center of Traditional Chinese and Modern Medicine, Hualien Tzu Chi Hospital, Buddhist Tzu Chi Medical Foundation, Hualien, Taiwan.; 3 School of Post-Baccalaureate Chinese Medicine, College of Medicine, Tzu Chi University, Hualien, Taiwan.; 4 Cardiovascular and Mitochondrial Related Disease Research Center, Hualien Tzu Chi Hospital, Buddhist Tzu Chi Medical Foundation, Hualien, Taiwan.; 5 Department of Psychiatry, Tzu Chi General Hospital, 707, Section 3, Chung-Yang Road, Hualien 97004, Taiwan.; 6 School of Medicine Tzu Chi University, 701, Section 3, Chung-Yang Road, Hualien 97004, Taiwan.; 7 Department of Emergency Medicine, China Medical University Hospital, Taichung, Taiwan.; 8 Division of Cardiovascular Medicine, Department of Internal Medicine, China Medical University Hospital, Taichung, Taiwan.; 9 Laboratory of Exercise Biochemistry, University of Taipei, Taiwan.; 10 Department of Biological Science and Technology, College of Life Sciences, China Medical University, Taichung, Taiwan.; 11 Ph.D. Program for Biotechnology Industry, China Medical University, Taichung, Taiwan.; 12 School of pharmacy, China Medical University, Taichung, Taiwan.; 13 Department of Biological Science and Technology, Asia University, Taichung, Taiwan.; 14 Center of General Education, Buddhist Tzu Chi Medical Foundation, Tzu Chi University of Science and Technology, Hualien 970, Taiwan.; 15 Graduate Institute of Biomedical Sciences, China Medical University, Taichung 404, Taiwan.; 16 Department of Medical Research, China Medical University Hospital, China Medical University, Taichung 404, Taiwan.

## Abstract

Age-related structural and functional changes in the kidney can eventually lead to development of chronic kidney disease, which is one of the leading causes of mortality among elderly people. For effective management of age-related kidney complications, it is important to identify new therapeutic interventions with minimal side-effects. The present study was designed to evaluate the synergistic effect of a traditional Chinese herb, Alpinate Oxyphyllae Fructus (AOF), and adipose-derived mesenchymal stem cells (ADMSCs) in ameliorating D-galactose (D-gal)-induced renal aging phenotypes in WKY rats. The study findings showed that D-gal-induced alteration in the kidney morphology was partly recovered by the AOF and ADMSC co-treatment. Moreover, the AOF and ADMSC co-treatment reduced the expression of proinflammatory mediators (NFkB, IL-6, and Cox2) and increased the expression of redox regulators (Nrf2 and HO-1) in the kidney, which were otherwise augmented by the D-gal treatment. Regarding kidney cell death, the AOF and ADMSC co-treatment was found to abolish the proapoptotic effects of D-gal by downregulating Bax and Bad expressions and inhibiting caspase 3 activation. Taken together, the study findings indicate that the AOF and ADMSC co-treatment protect the kidney from D-gal-induced aging by reducing cellular inflammation and oxidative stress and inhibiting renal cell death. This study can open up a new path toward developing novel therapeutic interventions using both AOF and ADMSC to effectively manage age-related renal deterioration.

## Introduction

In the past decade, the number of elderly population who are over the age of 65 years has increased significantly due to advanced medical facilities and improved lifestyle 1. Despite having an improved life expectancy, the elderly population is particularly susceptible to several age-related diseases, including cardiovascular and pulmonary disorders, renal problems, diabetes, gastrointestinal problems, osteoarthritis, neurodegenerative diseases, and even cancer 2. Thus, it is of prime importance to search for aging interventions that can delay the onset of age-related diseases, as well as subside the consequences of normal aging process, such as physical disabilities and functional limitations 3.

The application of mesenchymal stem cells (MSCs) as regenerative medicine has been increased tremendously in biomedical sciences. MSCs are potent candidates for cell-based therapies due to their unique properties of escaping immune surveillance and inhibiting inflammation after transplantation. Moreover, MSCs can be easily extracted from the bone marrow and adipose tissue, and differentiated into multiple organ-specific cell types 4. Given the ability of MSCs to repair damaged tissues and organs, several preclinical and clinical studies have been performed to evaluate the potency of MSC-based therapies in age-related diseases, because tissue degeneration is one of the major hallmarks of aging 5-8.

The risk of developing chronic kidney disease in elderly population is particularly high because of the increasing prevalence of other comorbid, predisposing risk factors, such as hypertension, diabetes, and cardiovascular disorders 9. Because of the significant anti-inflammatory effects, MSC-based therapies are considered to be very effective in treating kidney disorders wherein inflammation is one of the major contributing factors 10, 11. Several studies have been conducted to evaluate the effect of MSC-based treatments in various types of kidney disorders, such as acute kidney injury, focal segmental glomerulosclerosis, systemic lupus erythematosus, diabetic nephropathy, and kidney transplantation 12. Mechanistically, MSCs work as regenerative medicine by secreting a large number of bioactive molecules and extracellular vesicles that are collectively responsible for the therapeutic effects 13. However, the biological activity of MSCs can be drastically reduced in response to the challenging cellular microenvironment they encounter after transplantation 14, 15. In this context, MSC preconditioning using various external stimuli, such as hypoxia, pro-inflammatory mediators, growth factors, and 3D culture, is the best way to preserve and improve the therapeutic efficacy of MSCs 14.

Bioactive plant molecules or phytochemicals have become an attractive choice for treating many health complications particularly because of beneficial therapeutic properties, including anti-oxidative, anti-inflammatory, and tissue regenerative effects 14, 16. Moreover, a growing pool of evidence has suggested that phytochemicals can improve the basic characteristics of MSCs, including proliferation, differentiation, and migration 17.

Alpinate Oxyphyllae Fructus (AOF) is a Chinese herb used widely as traditional Chinese medicine for treating many health complications, including kidney, gastrointestinal, and cardiovascular disorders 18, 19. Bioactive components present in AOF extract, such as flavonoids, terpenes, essential oils, steroids, and diarylheptanoids, are known to possess several health benefits, including antioxidative, anti-inflammatory, anticancer, anti-diuretic, anti-diabetic, anti-diarrheal, anti-aging, and neuroprotective properties 20-23. Moreover, previous studies also indicated AOF can provide anti-aging effects in aging hearts by decreasing apoptosis and enhancing longevity gene expression 24. Interestingly, a recent study has shown that preconditioning of adipose-derived mesenchymal stem cells (ADMSCs) with AOF extract causes enrichment in ADMSC stemness, an increase in longevity, and improvement in stem cell homing 23. Moreover, AOF-preconditioned ADMSCs have been shown to protect cardiac cells from doxorubicin-induced cellular senescence. These hints suggested that AOF may provide beneficial effects in ADSC treated with aging renal.

Given the beneficial effect of AOF on the urinary system, as well as its ability to improve ADMSC properties, the present study was designed to evaluate the effect of AOF and ADMSC co-treatment on the kidneys of D-galactose-induced aging rats.

## Materials and Methods

### Alpinia extract

Alpinia was purchased from Shin-Long Pharmaceutical Company (Taiwan, ROC). For water extraction, 150 g of AOF was boiled in 600 ml of water for 2 h, followed by passing of the extract through Whatman filter paper at a reduced pressure. The filtrate was stored until become powder. The yield was approximately 7.4%. For preparing the stock solution, the powder was dissolved in water, and the solution was filter sterilized before administering to animals.

### Animals and experimental design

The entire experimental procedures and animal handling were supervised and approved by the China Medical University - Animal Care and Use Committee (IACUC) (No. 2016-172). The experiments were conducted on 18-week-old Wistar male rats that were purchased from the National Laboratory Animal Centre, Taiwan. The rats were maintained at room temperature of 23 ± 2°C under a dark/light cycle and were supplied with drinking water and rat chow ad libitum. After acclimatization for a couple of weeks, WKY rats were randomly divided into 6 groups (n = 4 per group): Control group, D-galactose (150 mg/kg/day) (D-gal) group, D-gal + AoF group, D-gal + ADMSC group, D-gal + AoF Oral + ADMSC group, and D-gal + AoF treated ADMSC group. For the treatments, AoF extract (100 mg/kg/day) was administered using oral gavage, and ADMSCs (10,000,000 cells) were administered intravenously. D-gal will treat with 6 weeks, after 6 weeks will provide treatment for 4 weeks, the rats were anesthetized and sacrificed by decapitation. The kidneys were harvested from the rats and immediately kept at -80°C.

### Hematoxylin-Eosin (H&E) Staining

The slides containing paraffin-embedded kidney sections were de-waxed by immersing in xylene (x 3) and dehydrated by dipping in ethanol gradients (100%, 95% and 75%). Then, the sections were incubated with hematoxylin staining solution for 5 min, followed by washing with double-distilled water (x 3) and submerging in 85% alcohol for 2 min. Next, the sections were incubated with eosin solution for 5 min, followed by submerging in 100% alcohol for 5 min and xylene for 1 min (x 2). The tissue sections were observed under microscope (Olympus Microscope), and the images were obtained.

### PAS staining

The slides were de-paraffinized and dehydrated with ethanol. Then they were immersed in PAS solution (10 min). After incubation, slides were washed using water (X 4). Then the samples were treated with Schiff's solution an incubated for 15min. After washing, the samples were incubated with Hematoxylin (3 min). Again the samples were washed with water, then for few seconds samples were kept with Bluing reagent. Finally, after washing, Light Green Solution was added for 2 min. Samples were dehydrated again with alcohol. The tissue sections were observed under microscope (Olympus Microscope), and the images were documented.

### TUNEL assay

The tissue sections were incubated with xylene, then the slides were washed in a series of alcohol. Slides were kept in proteinase K (2 μg/ml), followed by phosphate-buffered saline washing. Then they were treated with permeabilization solution and incubated in blocking buffer, and then washed with PBS. The terminal deoxynucleotidyl transferase and fluorescein isothiocyanate-dUTP apoptosis detection kit (Roche Applied Science, Indianapolis, IN, USA) was utilized for 60 min at 37 °C. Bright-green light at 450-500 nm was used to detect the number of apoptotic nuclei. The tissue sections were also stained with 0.1 mg/ml 4′,6-diamidino-2-phenylindole (DAPI) and detected by UV light at 340-380 nm. Photomicrographs were obtained using an Olympus® CKX53 microscope. All counts were performed by at least three independent individuals in a blinded manner.

### Tissue extraction

The kidney tissue (100 mg) was excised, washed with 1X PBS (x 3), and homogenized in 1 ml of RIPA buffer using tissue homogenizer. The lysate was kept on ice for 20 min, followed by centrifugation at 1,200 rpm for 5 min (4°C). The clear upper layer of the lysate was collected. After a few seconds of vortex, the supernatant was centrifuged at 13,000 rpm for 30 min (4°C), and the clear supernatant was stored for experimental usage.

### Western blotting

The protein samples were extracted from the harvested kidney tissues and quantified using Lowry's method. An appropriate amount of protein sample (40µg/lane) was mixed with 5X loading dye and boiled for 10 min at 95°C. The samples were vortexed, centrifuged, and loaded on the gel for SDS-PAGE analysis (current: 90 V; run time: 2 h). After the separation, the protein samples were transferred to a PVDF membrane, followed by blocking with 2.5% BSA for 1 h. Next, the membrane was rinsed in washing buffer and incubated with indicated primary antibodies overnight at 4°C. After washing with wash buffer (X3), the membrane was incubated with respective secondary antibodies for 1 h and washed with TBST. The immunoblot was developed with chemiluminescence ECL, and the bands were observed in LAS-4000 mini (GE Healthcare). The changes in protein expressions were compared using 'Image J' software.

## Results

### Synergistic effect of AOF and ADMSC cotreatment on D-galactose-induced aging kidneys

To investigate the effect of AOF and ADMSC cotreatment on aging kidney morphology, 18-week-old WKY male rats were treated with D-galactose (150 mg/kg/day) to induce aging phenotypes. After induction of aging, the rats were further treated with AOF extract alone (100 mg/kg/day); ADMSC cells alone (10,000,000 cells); AOF + ADMSC; and AOF-preconditioned ADMSC. The untreated rats were used as negative control.

**Figure 1:**
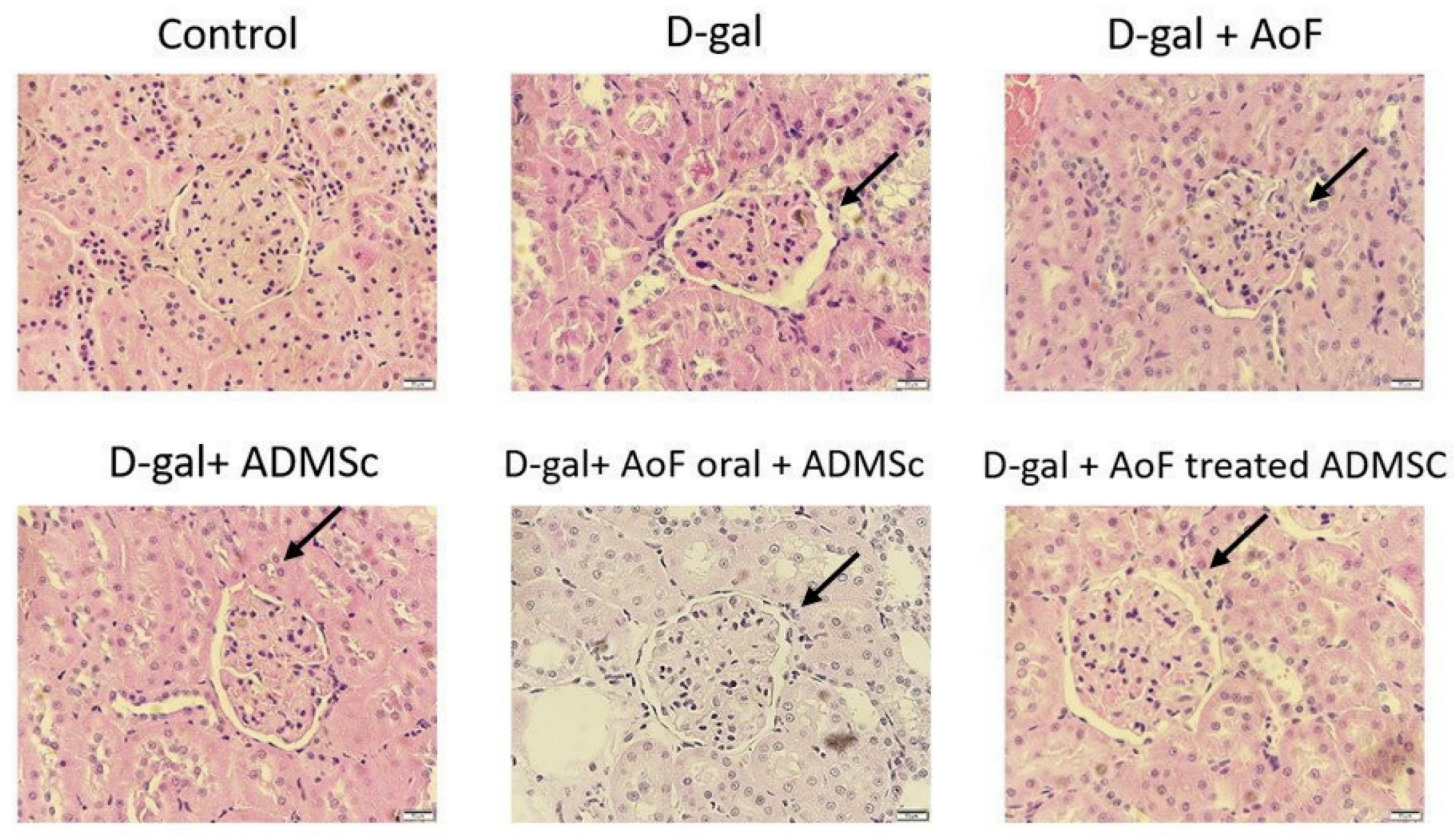
AOF and ADMSC decrease aging-induced kidney injury. Kidney injury evaluated by tissue section and H&E staining. D-gal induced renal aging significant showed the organization is not neatly arranged (arrow site). After treated with AOF and ADMSc significant decrease the arranged sites compared with D-gal group (arrow site).

The kidney tissues excised from the aging rats were subjected to hematoxylin-eosin staining to investigate effects of AOF and ADMSC in ameliorating the histological changes induced by D-galactose. As observed in Figure 1, D-galactose treatment caused significantly increased Bowman's space and altered shape and size of glomeruli. These findings clearly indicate D-galactose-induced onset of glomerular aging. Interestingly, it was observed that cotreatment with AOF and ADMSC was capable of moderately ameliorating the aging-induced renal phenotypes.

**Figure 2:**
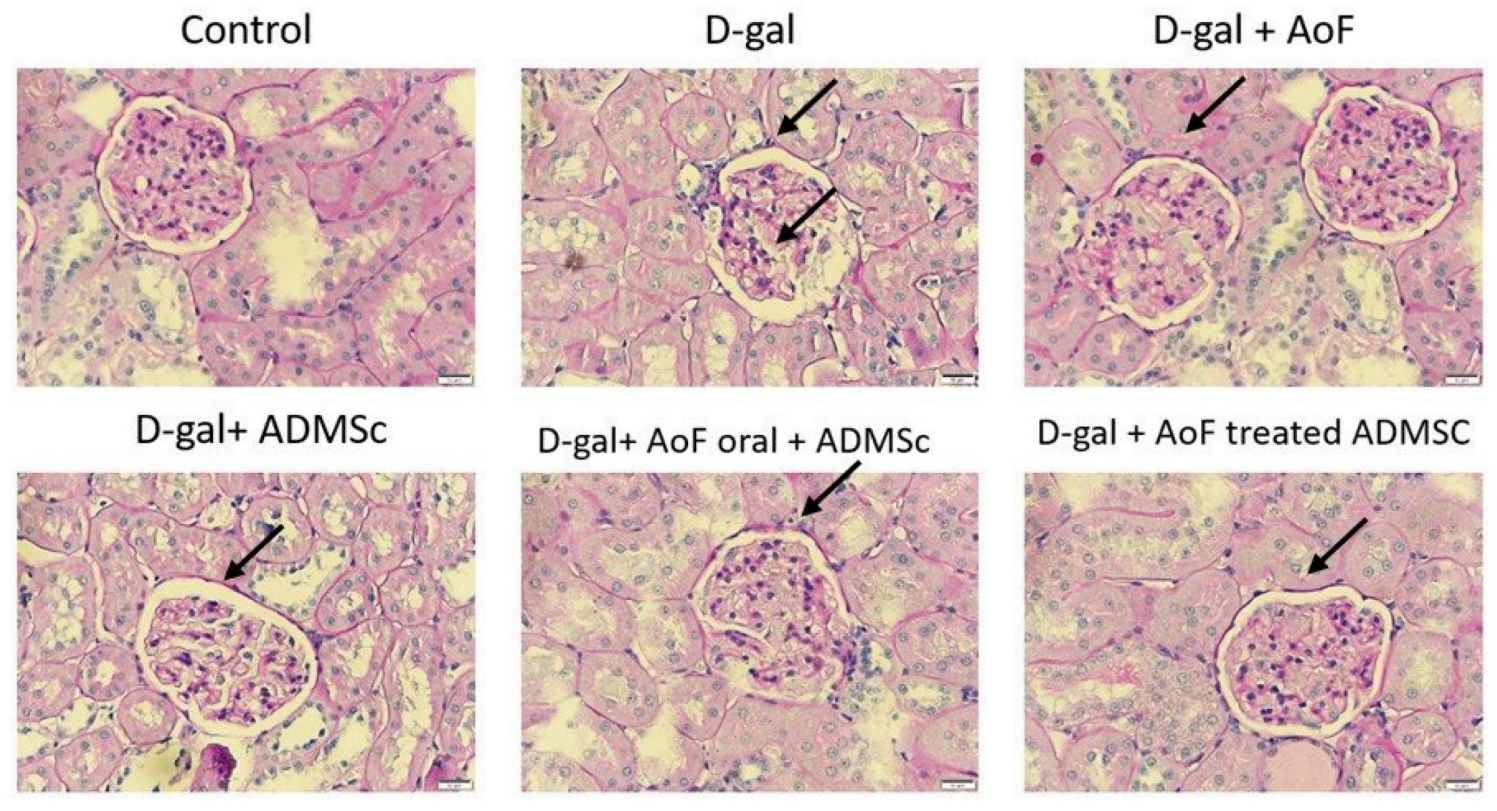
AOF and ADMSC prevent D-gal induce kidney aging. Kidney injury evaluated by tissue section and PAS staining (red color). D-gal induced kidney aging (arrow site), treated with AOF or ADMSc or co-treated both can decreased aging in kidney arrow site.

Next, PAS staining was performed to further investigate the renal protective effects of AOF and ADMSC. As observed in Figure 2, D-gal treatment caused thickening of the capillary wall, which was recovered, at least in part, by AOF and ADMSC cotreatment. Taken together, these findings clearly depict renal protective effects of AOF and ADMSC cotreatment on D-gal-induced aging kidneys.

### Protective effects of AOF and ADMSC cotreatment on oxidative and inflammatory stress markers in D-galactose-induced aging kidneys

**Figure 3:**
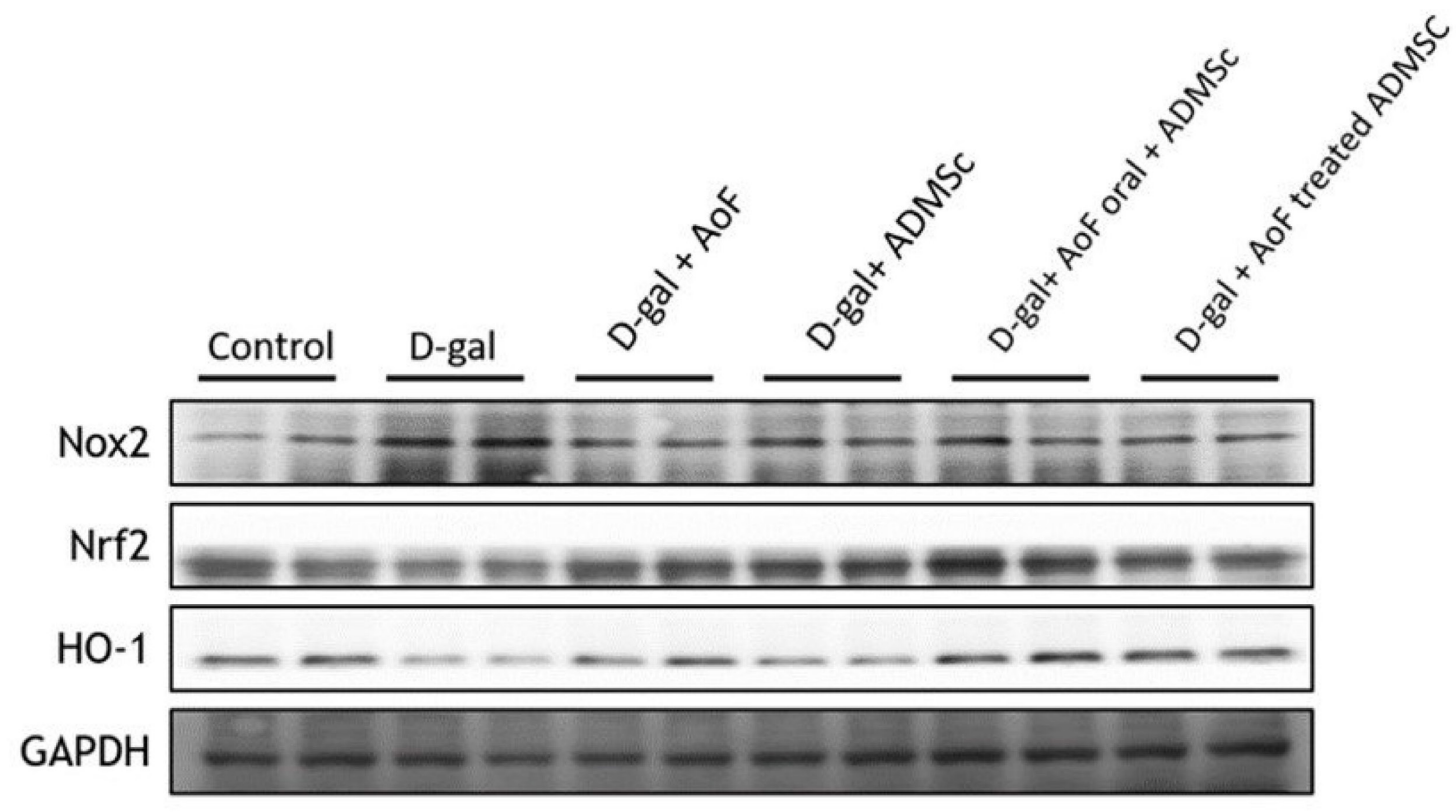
D-gal induce ROS injury by targeting Nrf2 molecular pathway. D-gal significantly increased Nox2 expression and decreased ROS-related molecular proteins Nrf2 an d HO-1 expression. Treatment with AOD or ADMSC significantly decreased injury from D-gal, analysis by western blotting.

Since oxidative stress and inflammation play crucial roles in age-related renal pathologies 25, we next investigated the effects of AOF and ADMSC cotreatment on biomarkers of oxidative stress and inflammation in D-gal-induced aging kidneys. To evaluate cellular oxidative stress, the protein expressions of NADPH oxidase (NOX2), nuclear factor erythroid 2-related factor 2 (Nrf2), and heme oxygenase-1 (HO-1) were analyzed using immunoblotting method. As observed in Figure 3, D-gal treatment caused significant induction of oxidative stress, which was characterized by increased NOX2 expression and decreased Nrf2 and HO-1 expressions. Interestingly, AOF and ADMSC cotreatment was found to restore the redox homeostasis by enhancing Nrf2 and HO-1 expressions.

**Figure 4:**
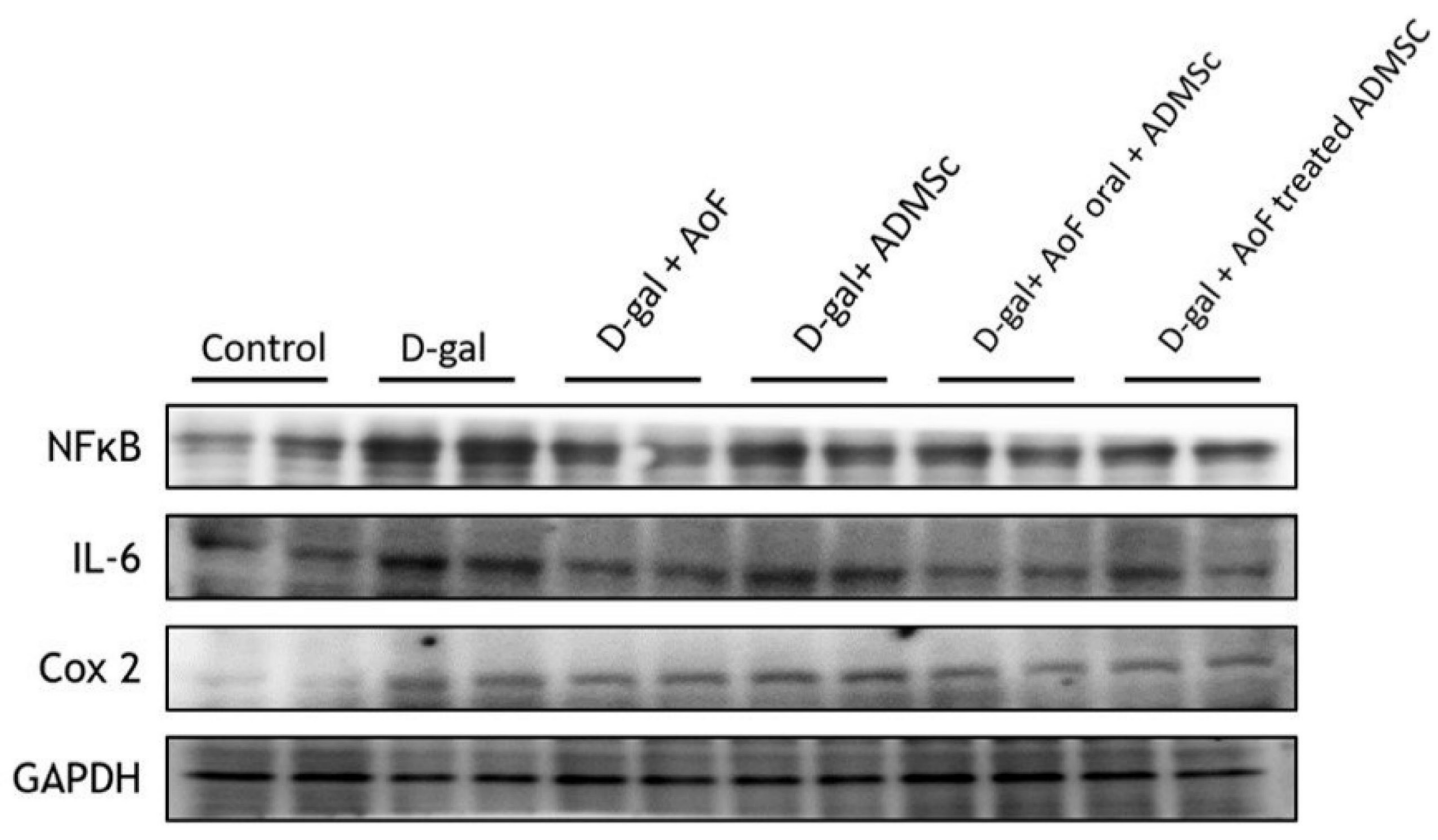
AOF and ADMSC decrease D-gal induced inflammation in kidney. D-gal increased inflammation-related protein, and after treatment with AOF and ADMSC significantly decreased inflammation-related protein factor expression, analysis by western blotting.

To investigate cellular inflammation, the protein expressions of pro-inflammatory mediators including nuclear factor NFkB, interleukin-6 (IL-6), and cyclooxygenase 2 (Cox2) were analyzed using kidney tissue extracts. As observed in Figure 4, D-gal-induced induction of inflammatory microenvironment in aging kidney tissues was well evidenced by the increased expressions of NFkB, IL-6, and Cox2. However, the cotreatment with AOF and ADMSC was found to considerably ameliorate renal inflammation by reducing the expressions of pro-inflammatory mediators.

### Synergistic effect of AOF and ADMSC cotreatment on renal cell death

**Figure 5:**
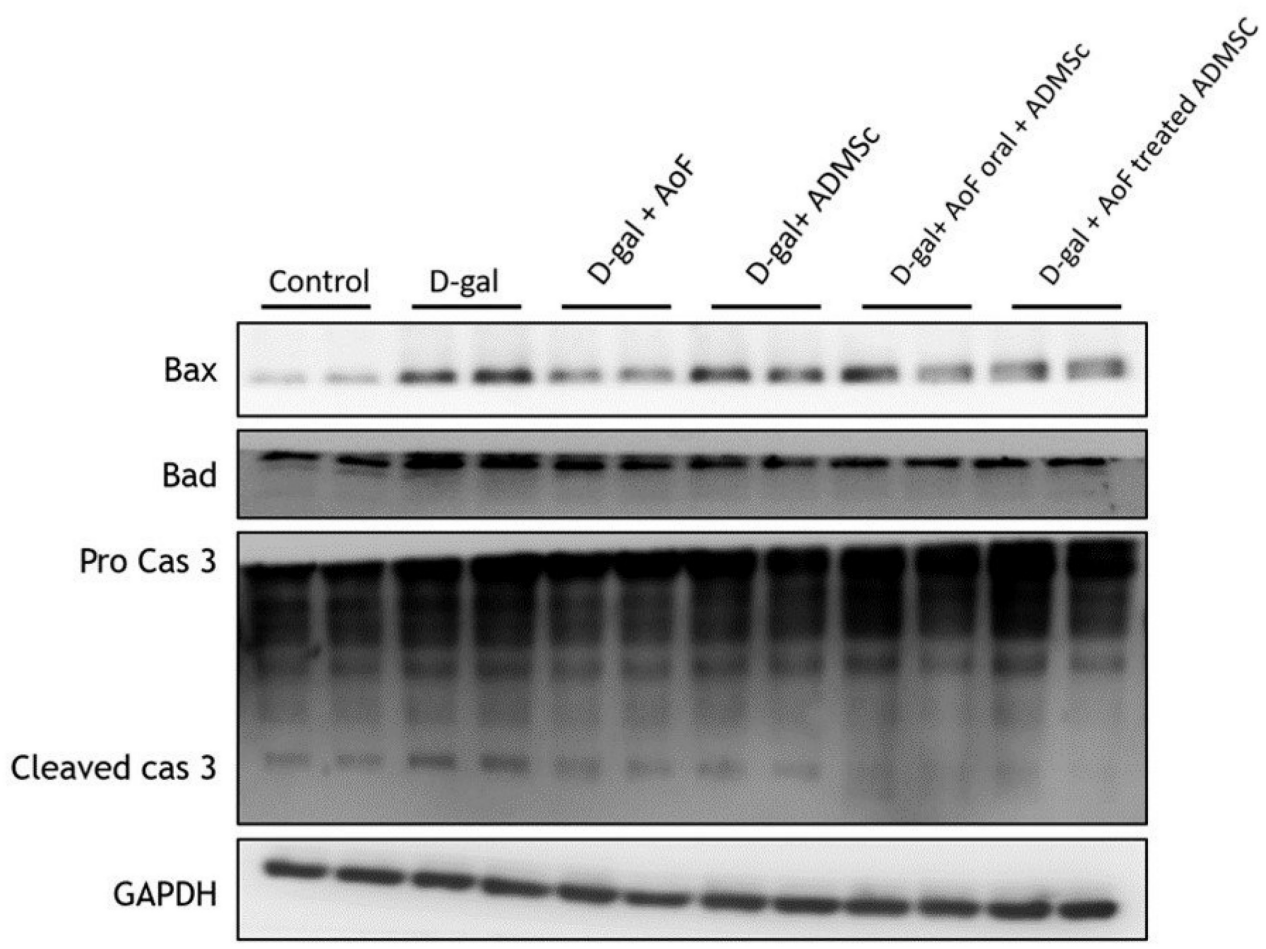
AOF and ADMSC decreased D-gal-induced apoptosis in kidney. Treated with AOF, ADMSC in Rat significantly decreased D-gal-induced apoptosis-related protein expression.

To investigate the effects of AOF and ADMSC on renal cell apoptosis, the protein expressions of intrinsic (mitochondria-mediated) apoptotic pathway components including Bax, Bad, and caspase 3 were analyzed using kidney tissue extracts. Mechanistically, induction of BH3 domain-containing pro-apoptotic protein (Bax and Bad) expression causes release of cytochrome c from mitochondria to the cytosol, leading to activation of caspase 3 and induction of apoptosis 26. As observed in Figure 5, D-gal-induced increased expression of Bax, Bad, and caspase 3 was counterbalanced by the administration of AOF and ADMSC, indicating anti-apoptotic effects of AOF and ADMSC on aging renal cells.

**Figure 6:**
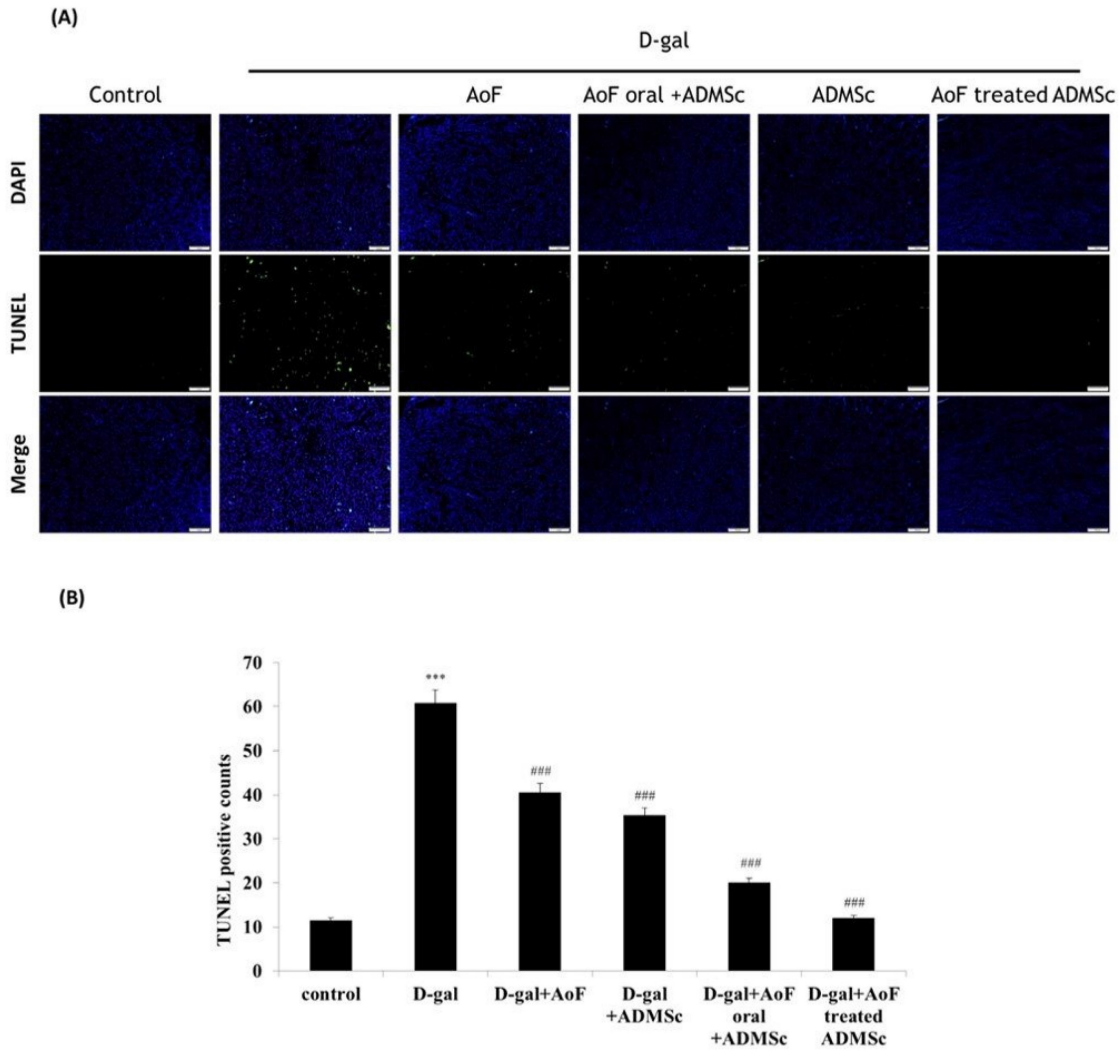
AOF and ADMSC provide protective effects in aging kidney. (A) D-gal induced kidney aging and cell death (green spots). After treatment with AOF or ADMSC in aging rats, significant decrease in cell death, analysis on tissue section by TUNEL assay. (B) Quantification of the TUNEL-positive cells by Image J software.

Similarly, the TUNEL assay data showed that the number of apoptotic cells reduced significantly upon administration of AOF and ADMSC, which were otherwise increased by D-gal treatment (Figure 6). Taken together, these observations clearly indicate the health benefits of AOF and ADMSC co-treatment on D-gal-induced renal aging phenotypes.

## Discussion

Structural and functional alterations in the kidney are common consequences of aging. These changes grossly include reduced functionality of kidney blood vessels, increased number and size of nephrons, thickened glomerular basement membrane, reduced glomerular filtration rate, interstitial fibrosis, and tubular atrophy 27, 28. The age-related structural alterations are commonly defined as nephrosclerosis, which is developed due to nonmalignant hypertension. Importantly, nephrosclerosis along with kidney dysfunction can make elderly people more susceptible to acute kidney injury and chronic kidney disease 29. Thus, it is of prime importance to identify novel therapeutic interventions with minimal side-effects to manage age-related kidney deterioration.

Given the significant impact of MSC-based therapies in treating kidney disorders 10, the present study was designed to evaluate the effect of a traditional Chinese herb, AOF, in the potentiation of MSC functions, as well as to investigate the effects of AOF and ADMSC co-treatment in ameliorating age-related renal changes.

We used D-gal for the induction of aging phenotypes, and our data showed that D-gal caused age-related structural changes in the kidneys, such as increased Bowman's space and altered shape and size of glomeruli (Figure 1). Although treatment with AOF or ADMSC alone showed ameliorating effects on the aging kidney tissue, the maximum benefit was observed with AOF and ADMSC co-treatment (Figure 1). These findings suggested the possible effect of AOF extract in improving ADMSC characteristics, which is in line with a previous study indicating AOF pretreatment induced improvement in ADMSC longevity and homing ability 23. Further analysis of the kidney morphology using PAS staining revealed that AOF and ADMSC co-treatment was capable of restoring D-gal-induced thickening of the capillary wall (Figure 2).

Similar to our findings, the renally protective role of AOF has been observed previously 30, 31. By reducing the oxidative stress level and modulating PTEN expression, AOF extract has been found to improve kidney functions in diabetic mice 32. Regarding renal morphological changes, AOF extract has been shown to prevent tubular necrosis in rat models of ischemia/reperfusion-induced acute renal failure 22.

Since inflammation and oxidative stress are two major predisposing factors for kidney disorders, we investigated several oxidative stress and inflammatory biomarkers in the present study. Our western blot data showed that D-gal treatment caused induction of NOX2 expression, which was accompanied with reduced Nrf2 and HO-1 expressions (Figure 3). This indicates that D-gal-induced renal aging is associated with induction of oxidative stress. However, co-treatment with AOF and ADMSC was found to reverse the D-gal effects by reducing NOX2 expression and increasing Nrf2 and HO-1 expressions in the kidney tissues (Figure 3). Regarding inflammation, we observed that co-treatment with AOF and ADMSC diminished renal inflammation by reducing the expressions of pro-inflammatory biomarkers including NFkB, IL-6, and Cox2, which were otherwise increased after the D-gal treatment (Figure 4).

Previous studies concerning health benefits of AOF extracts have also highlighted the antioxidant and anti-inflammatory properties of AOF 33, 34. In a rat model of hypoxia-induced enuresis, treatment with AOF extract has been found to reduce urine output and increase bladder leak point pressure by increasing superoxide dismutase (SOD) activity, decreasing malondialdehyde (MDA) level, and regulating purinergic, muscarinic, and beta-adrenergic receptors 35. Regarding inflammation, AOF extract has been shown to reduce the symptom severity of encephalomyelitis in mice by decreasing the infiltration of pro-inflammatory mediators in the spinal cord and regulating Th1/Th17 response 21. Moreover, research also indicated AOF increased the longevity related genes such as SIRT1 molecular pathway to protect against aging injury in heart 24. These results and our data suggested that AOF can provide a strong anti-aging effect in many organs.

The antioxidative and anti-inflammatory benefits of MSC are also we-evidenced in the literature 36. Co-culture of MSCs with airway smooth muscles has been found to reduce mitochondrial ROS production, maintain mitochondrial membrane potential, and prevent cell death 37. Moreover, MSC has been found to attenuate adriamycin-induced nephropathy by reducing oxidative stress, downregulating NFkB expression, and reducing inflammatory cytokine production 38. In line with these observations, our findings reveal that the co-treatment with AOF and ADMSC synergistically ameliorates D-gal-induced renal aging by reducing inflammation and maintaining redox homeostasis.

Furthermore, we observed that the treatment with D-gal increased the expressions of Bax and Bad and activated caspase 3 (Figure 5), which was accompanied with increased numbers of apoptotic cells (Figure 6). Interestingly, AOF and ADMSC co-treatment was found to completely nullify the D-gal effects (Figure 5 and 6). These findings indicate that AOF and ADMSC collectively prevent D-gal-induced renal aging by reducing oxidative stress- and inflammation-induced renal cell death.

The present study observations are supported by previous studies showing the anti-apoptotic effects of MSCs against hypoxia-induced alveolar epithelial cell death 39. Moreover, exosomes derived from MSC have been shown to reduce ER stress-induced apoptosis of nucleus pulposus cells 40. Interestingly, ADMSC pretreated with AOF extract has been shown to protect hepatic cells from doxorubicin-induced senescence and aging.

## Conclusion

The present study was designed to evaluate the synergistic effect of a traditional Chinese herb, AOF, and ADMSC in ameliorating D-gal-induced renal aging phenotypes. The study findings reveal that the co-treatment with AOF and ADMSC is capable of restoring D-gal-induced alterations in renal morphology. Mechanistically, AOF and ADMSC collectively ameliorate D-gal-induced renal aging by reducing the expression of inflammatory mediators (NFkB, IL-6, and Cox 2); increasing the expression of redox regulators (Nrf2 and HO-1); and preventing renal cell death. The present study findings can open up a new path toward developing novel therapeutic interventions using both AOF and ADMSC to effectively manage age-related renal deterioration.
